# Early upregulation of AR and steroidogenesis enzyme expression after 3 months of androgen-deprivation therapy

**DOI:** 10.1186/s12894-020-00627-0

**Published:** 2020-06-19

**Authors:** Agus Rizal A. H. Hamid, Harun W. Kusuma Putra, Ningrum Paramita Sari, Putri Diana, Saras Serani Sesari, Eka Novita, Fajar Lamhot Gultom, Meilania Saraswati, Budiana Tanurahardja, Asmarinah, Rainy Umbas, Chaidir A. Mochtar

**Affiliations:** 1 Department of Urology, CiptoMangunkusumo Hospital - Faculty of Medicine Universitas Indonesia, Jl. Diponegoro No.71, RW.5, Kenari, Senen, RW.5, Kenari, Senen, Kota Jakarta Pusat, Daerah Khusus Ibukota Jakarta, 10430 Indonesia; 2 Department of Biology, CiptoMangunkusumo Hospital - Faculty of Medicine Universitas Indonesia, Jl. Diponegoro No.71, RW.5, Kenari, Senen, RW.5, Kenari, Senen, Kota Jakarta Pusat, Daerah Khusus Ibukota Jakarta, Indonesia; 3 Department of Pathology Anatomy, CiptoMangunkusumo Hospital - Faculty of Medicine Universitas Indonesia, Jl. Diponegoro No.71, RW.5, Kenari, Senen, RW.5, Kenari, Senen, Kota Jakarta Pusat, Daerah Khusus Ibukota Jakarta, Indonesia

**Keywords:** Androgen receptor, Steroidogenic enzyme, AKR3C1 SRD5A1, SRD5A2, SRD5A3

## Abstract

**Background:**

Androgen deprivation therapy (ADT) is a standard treatment for advanced prostate cancer (PCa). However, PCa recurrence and progression rates during ADT are high. Until now, there has been no evidence regarding when progression begins. This study evaluated the gene expression of intraprostatic androgen receptor (AR) and steroidogenic enzymes in the early stages of ADT.

**Methods:**

Prostate tissue samples were taken from PCa patients with urinary retention who received ADT (ADT-PCa; *n* = 10) and were further subgrouped into ADT ≤12 months (*n* = 4) and ADT > 12 months (*n* = 6). The ADT-PCa tissues were then compared with BPH (*n* = 12) and primary (no treatment) PCa tissues (*n* = 16). mRNA for gene expression analysis of AR and steroidogenic enzymes was extracted from formalin-fixed paraffin embedded (FFPE) tissues and analyzed by real-time PCR. Protein expression was evaluated by immunohistochemistry with specific antibodies.

**Results:**

AR gene expression was higher in the ADT-PCa group than in the BPH or primary PCa group. Both the ADT ≤12 and > 12 months subgroups had significantly higher relative gene expression levels of AR (*p* < 0.01 and 0.03, respectively) than the primary PCa group. In the ADT-PCa group, AR protein expression showed an increasing trend in the ADT ≤12 months subgroup and was significantly elevated in the ADT > 12 months subgroup compared with the PCa group (100%; *p* < 0.01). Half (50%) of the patients in the ADT ≤12 months subgroup were found to have upregulation of AR, and one showed upregulation beginning at 3 months of ADT. A trend toward elevated relative gene expression of SRD5A3 was also apparent in the ADT groups.

**Conclusion:**

AR and steroidogenic enzymes are upregulated in ADT-PCa patients as early as 3 months, without PSA elevation. Steroidogenic enzymes, particularly SRD5A3, were also upregulated before PSA rose.

## Background

Over the years, there have been notable improvements in the development of prostate cancer (PCa) treatment, resulting in better prognoses [1]. However, 40% of patients with early PCa are still at risk of disease progression to a more advanced stage [1] and thus need androgen deprivation therapy (ADT), the current standard palliative treatment [2]. Although ADT may prolong overall survival [3], prolong the occurence of metastasis [4], and decrease PSA levels [5], these effects are only temporary. The disease often progresses further after 2–3 years of treatment [6]. This form of late-stage PCa is called castration-resistant prostate cancer (CRPC), as PCa cells have evolved and become capable of both hormone-dependent and hormone-independent cell proliferation and survival [7].

The existence of CRPC has motivated an increased focus on the disease. The androgen receptor (AR) signaling pathway is an important mechanism of PCa and plays a key role in PCa progression [8–10]. The known mechanisms of resistance include AR overexpression, AR gene amplification, AR hypersensitivity, AR mutations, AR variants (ARVs), androgen-independent AR activation, and intratumoral and alternative androgen production [8–10]. Furthermore, data have suggested that the increased level of intraprostatic AR can lead to disease progression [10]. Other downstream steroidogenic enzymes within the AR signaling pathway, 5α-reductase and AKR1C3, have also become targets of interest [7–12]. These enzymes are responsible for the conversion of androgens into dihydrotestosterone (DHT), which is needed by PCa cells [11]. Currently, there are three identified isozymes of SRD5A encoded by 3 different genes: SRD5A1, SRD5A2, and SRD5A3 [11]. AKR1C3, SRD5A1 and SRDA2 have been extensively studied [7–12], and marked changes in their levels were reported to occur during the course of PCa development and progression [7–12]. SRD5A1 and SRD5A3 are associated with androgen response elements (AREs) that promote the transcription of target genes, leading to cell homeostasis, angiogenesis, differentiation, and apoptosis [13]. It is also believed that the two isozymes play important roles in androgen production in prostate cells [11]. On the other hand, the role of SRD5A3 is still obscure, although associations with DHT production and AR activation have been suggested [14]. Based on these findings, AR, AKR1C3, and 5α-reductase are indicative of PCa progression, as differences in their levels between malignant and nonmalignant cells have been documented. However, changes in the levels of these genes following ADT treatment have never been reported, although they have been associated with further disease progression and recurrence.

Currently, no study has reported the start of disease progression after ADT. This study hypothesized that progression of the disease would likely occur within a short time interval after commencement of ADT. Therefore, this study evaluated the early effects of ADT by investigating the levels of AR and steroidogenic enzymes as an indicator of disease progression. To this end, we compared the levels of these factors within the intraprostatic cells of benign prostatic hyperplasia (BPH) and primary PCa cases to those of PCa cases after ADT treatment.

## Methods

### Samples

Formalin-fixed paraffin embedded (FFPE) prostate tissue was obtained from the Department of Pathology Anatomy, Cipto Mangunkusumo General Hospital (RSCM). The study included three groups of patients: 1) the BPH group (*n* = 12), 2) the primary PCa group (*n* = 16, sampled from 2009 to 2015), and 3) the PCa after ADT (ADT-PCa) group (*n* = 10). The sample retrieval method for BPH and primary PCa are through biopsy, TUR-P, or Radical prostatectomy, while ADT-PCa tissues were taken primarily from TUR-P. The ADT-PCa group was categorized as the ADT ≤12 months and ADT > 12 months subgroups based on the duration of ADT. All use of specimens was in compliance with the guidelines of the local review board and Ethics Committee 520/UN2, F1/ETIK/2015, and prostatic tissues were obtained with patient consent at the time of the procedure.

### RNA extraction from FFPE tissue

Paraffinization was performed by prior isolation of the RNA using a High Pure RNA Paraffin Kit (Roche, Germany). The sample used was FFPE prostate tissues, which were cut to a thickness of 5–10 μM from each tissue sample from each group (BPH, primary PCa, and ADT-PCa). DNA extraction was performed from the FFPE samples. Total RNA was extracted in 50 μl and stored in a − 80 °C freezer.

### Quantitative real-time PCR

Quantification of gene expression (by qPCR) was performed for AR, AKR1C3, SRD5A1, SRD5A2, and SRD5A3 using a QuantiTect SYBR Green RT-PCR kit. The 18S gene was the housekeeping gene used as the internal control due to its constitutive expression across tissues and cells. qPCR mix (2x QuantiTect SYBR Green RT-PCR Mix, Forward Primer, Reverse Primer, free water nuclease) was used to set up the reactions, and 2 μL of the cDNA sample was used as the template. The specific primers used in this study are shown in Table 1. Measurements were performed using the Mx3000P (Agilent) tool with an amplification program. The cycle threshold (Ct) was obtained by using MxPro (Agilent) software. Relative gene expression was measured by using the Livak method, also known as the delta threshold cycle (ΔΔCt).

**Table 1:** Primers for rtPCR

Gene	Forward	Reverse
AR	CAT TGA GCC AGG TGT AGT GT	CCA GTT CAT TGA GGC TAG AGA G
AKR1C3	TGC AGG TTT TTG AGT TCC AGT	TGG CTA GCA AAA CTA TCA CGT T
SRD5A1	ACG GGC ATC GGT GCT TAA T	CCA ACA GTG GCA TAG GCT TTC
SRD5A2	GGAGTCCTTCAAGGCTACTATCT	CACCCAAGCTAAACCGTATGT
SRD5A3	GTC ATC TGC CCA TCA GTA TAA	GAA TGA CCA CTC CTG CTT TAT
18 S	AAA CGG CTA CCA CAT CCA AG	CCT CCA ATG GAT CCT CGT TA

### Immunohistochemistry

Immunohistochemistry was performed by using the FFPE preparation. Each sample was cut into 4-μm sections, warmed at 58 °C, and deparaffinized using xylol I, II, and III. The specimens were rehydrated in a 100, 96, and 80% graded ethanol series and were then subjected to heat-induced epitope retrieval (HIER) at pH 6 in a pressure boiler at 125 °C and cooled at 90 °C. A dual endogenous enzyme block was performed using 0.3% H_2_O_2_ and 95% ethanol. Staining for analysis of the expression of AR, AKR1C3 and steroidogenic enzymes SRD5A1, SRD5A2, and SRD5A3 was performed using antibodies obtained from Sigma Aldrich (St Louis, Missouri) [15]: mouse monoclonal anti-AR (WH0000367M1) [15, 16], mouse monoclonal anti-AKR1C3 (A6229) [15, 17], rabbit polyclonal anti-SRD5A1 (HPA051402) [15], rabbit polyclonal anti-SRD5A2 (SAB2105567) [15], and rabbit polyclonal anti-SRD5A3 (HPA027006) [15, 18]. All of the antibodies were validated, referenced (Human Protein Atlas project) and peer-reviewed and are widely used in the immunohistochemistry research field, as referenced by Sigma Aldrich. Additional information is provided in Supplementary Table 1.

Tissues were incubated with an HRP-labeled polymer followed by chromogenization in DAB (substrate DAB: chromogen DAB was 1:20) using the DAKO autostainer Kit. The specimens were then counterstained using hematoxylin and dehydrated (100, 80, 96%), cleared (xylol I, II, III), and mounted onto slides with coverglass.

Slides were assessed visually by three examiners who were blinded to the patient groups, and the results were confirmed by an experienced pathologist. Microscopic semiquantitative examination was performed on 10 random fields per specimen containing a minimum of 500 cells using ImageJ (Research Service Branch, NIH.gov) at 400× magnification. Protein expression was considered positive if the cells staining was brownish in the cytoplasm or if the nuclei had a granular pattern.

### H-score calculation to measure protein expression

Positively stained epithelial cells were then quantified using a semiquantitative scoring method of 0, + 1, + 2, and + 3 with expression scores for each cell. A score of + 1 was defined as low positive, + 2 as moderate positive, and + 3 as high positive. Quantification of protein expression was performed by measuring the H-score. The H-score was measured using the following formula:

$$ \left[\left(1\ \mathrm{x}\%+1\ \mathrm{cells}\right)+\left(2\ \mathrm{x}\%+2\ \mathrm{cells}\right)+\left(3\ \mathrm{x}\%+3\ \mathrm{cells}\right)\right] $$

The mean H-score of the BPH group was set as the cut-off to determine which samples showed upregulated or downregulated protein expressionand the results were further analyzed for statistical comparisons. The percentage of samples with upregulation is presented

### Statistical analysis

Statistical analysis was performed using GraphPad Prism 7 (La Jolla, CA). The Mann-Whitney U test was used to compare gene expression among the groups as follows: ADT versus BPH and ADT versus PCa. *P* < 0.05 was defined as statistically significant. Fisher’s exact test was used for comparisons of protein expression between specified groups and the associations with clinicopathological parameters.

## Results

### Sample characteristics

From 2007 to 2015, we were able to evaluate 10 patients who still had urinary retention after ADT treatment (ADT-PCa group), and their mean age was 67.27 ± 9.69. The group was then subdivided into two groups: one received ADT for under 12 months (4 samples), with a median treatment time of 5 (3–9) months, and the other received ADT for more than 12 months (6 samples), with a median treatment time of 30 (15–70) months. The initial PSA levels were 93.3 (58.14–784.60) in the ADT ≤ 12 months group and 176.9 (104.23–200.40) in the ADT > 12 months group. The Gleason scores of most of the patients were high [8–10], and the T-stages and prostate volumes were comparable between the two groups. The ADT types used were similar and comparable between the two groups: in the ADT ≤ 12 months group, 50% of the patients underwent orchiectomy and 50% received LHRH agonist and antiandrogen, while in the ADT > 12 months group, these proportions were 67 and 33% respectively. We also compared these patients to the BPH group (12 samples) and primary PCa group (16 samples). The detailed characteristics of the patients are presented in Table 2. There was statistically significant difference of PSA level between the groups, with the highest PSA level found in the ADT > 12 months group.

**Table 2:** Basic characteristics of patients

	BPH ***n*** = 12	Prostate Cancer			***P***-Value^**c**^
		Primary PCa ***n*** = 16	ADT-PCa ***n*** = 10		
			Duration ≤ 12 Months ***n*** = 4	Duration > 12 Months ***n*** = 6	
**Age**	64.2 ± 8.6	63.4 ± 7.0	58.75 ± 3.4	72 ± 9.3	0.05
**Sample retrieval method**					
Biopsy	5 (71%)	5 (31%)	–	–	–
TUR-P	2 (29%)	5 (31%)	4 (100%)	6 (100%)	
Radical Prostatectomy	–	6 (38%)	–	–	
**Gleason Score**					0.76
< 7	–	4 (25%)	–	1 (17%)	
8–10	–	12 (75%)	4 (100%)	5 (83%)	
**T Staging**^**a**^					0.91
1	–	6 (37%)	–	–	
2	–	4 (25%)	2 (50%)	3 (50%)	
3	–	3 (19%)	–	1 (17%)	
4	–	3 (19%)	2 (50%)	2 (33%)	
**Lymphatic Involvements**	–	1 (7%)	NE	NE	–
**PSA**^**a**^	9.5 (1.4–36.7)	49.3 (4.6–600)	24.13 (10.1–303.2)	203.2 (10.2–689)	0.01*
**Prostate volume**^**a**^	51.6 (28–73.6)	43.6 (13.4–80.5)	30.5 (28.6–36.68)	39.7 (27.6–94.2)	0.39
**ADT Type**					0.76
Orchyde	–	–	2 (50%)	4 (67%)	
LHRH agonist + antiandrogen	–	–	2 (50%)	2 (33%)	
**ADT Treatment Duration**	–	–	5 (3–9)	30 (15–70)	**–**

*N.E* not evaluated

^a^ PSA and prostate volume are presented in median (min-max)

^b^ Sample retrieval method, T staging, and Lymphatic involvements are presented as percentages

^c^ Statistical significance was measured by comparing Primary PCa, ADT-PCa ≤ 12 months and > 12 months using Pearson chi square for Gleason score, and ADT Type; ANOVA for age; Kurskall Wallis test for T-stage, PSA level, and Prostate Volume

^d^ ADT treatment duration is presented in months

^*^P-value < 0.05 is significant

### Relative gene expression

**Fig. 1:**
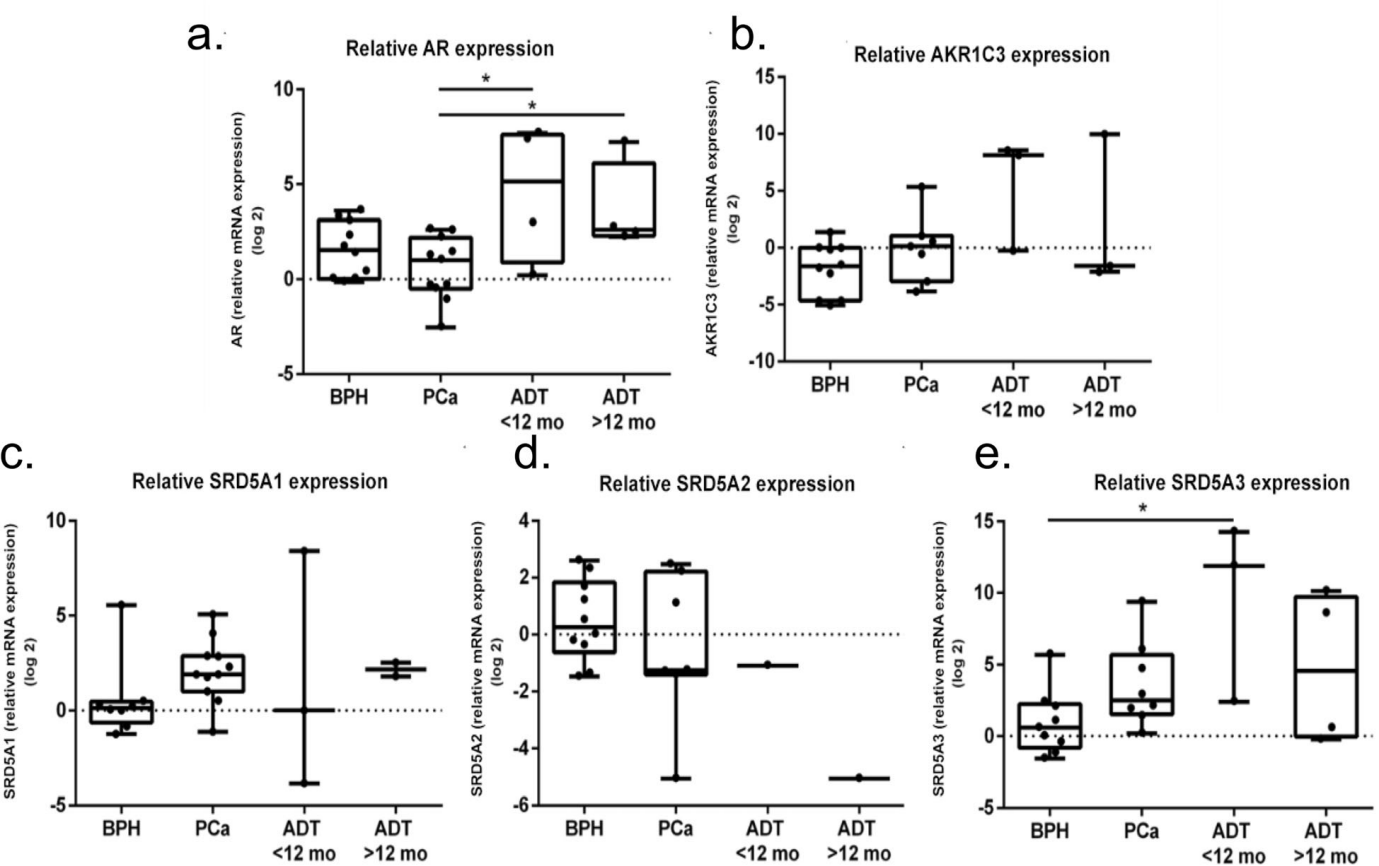
Gene expression analysis. Analysis of the relative expression of five different genes was performed by quantification of real-time one-step RT-PCR using the Mx3000P (Agilent) tool in tissue samples of the BPH group (*n* = 12), primary PCa group (*n* = 13), ADT ≤12 months group (*n* = 4) and ADT > 12 months group (*n* = 6). The included AR (**a**), AKR1C3 (**b**), SRD5A1 (**c**), SRD5A2 (**d**), and SRD5A3 (**e**). Statistical analysis was performed by using the Mann-Whitney test, comparing each group to another in a paired manner. * *p* < 0.05 and ** *p* < 0.01. Standard errors of the means are indicated by bars. **a**-**e** All experiments were performed at least two times

AR gene expression was higher in the ADT-PCa group than in the BPH (median difference of 1.1; *p*-value 0.05) and primary PCa (median difference of 1.83; *p* < 0.01) groups. Patients with ADT ≤12 months had the highest relative AR gene expression (median 5.14). AKR1C3 showed an increasing trend in the ADT-PCa subgroups compared with the BPH and primary PCa groups (Fig. 1).

There were no significant differences in the gene expression of SRD5A1 or SRD5A2 between the groups. However, a decreasing trend in SRD5A2 gene expression was noted (Fig. 1). In contrast, an overall increasing trend in SRD5A3 expression was observed between the BPH and ADT-PCa subgroups, with the ADT ≤12 months subgroup having the highest relative SRD5A3 expression (median difference of 9.39 in ADT ≤12 months vs the primary PCa group, *p* = 0.13; median difference of 11.3 in the ADT ≤12 months group vs the BPH group, *p* = 0.02) (Fig. 1).

### Protein expression by immunohistochemistry

**Fig. 2:**
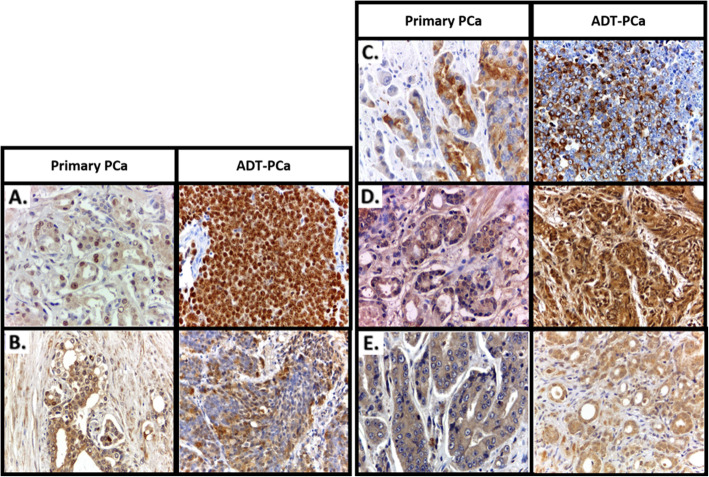
Immunohistochemistry of prostate cancer tissue stained for AR, AKR1C3, SRD5A1, SRD5A2, and SRD5A3. Comparison between the protein expression of AR and intraprostatic steroidogenic enzymes in the prostatic cells of the primary PCa and ADT-PCa groups: **a** AR, **b** AKR1C3, **c** SRD5A1, **d** SRD5A2, and **e** SRD5A3. Images were taken of sliced FFPE samples of patients obtained between 2009 and 2014 in RSCM. Images were captured at 400.000× magnification

AR and AKR1C3 expression was found in both the nucleus and cytoplasm, while the protein expression of SRD5A1, SRD5A2, and SRD5A3 was found predominantly in the cytoplasm (Fig. 2). Upregulation of protein expression is described in Table 3. AR protein expression in the ADT-PCa group was increased by 80% compared with that in the primary PCa group (*p* < 0.01), showing an increasing trend in the ADT ≤12 months subgroup compared with the PCa group (*p* = 0.2), and AR protein expression was significantly higher in the ADT > 12 months subgroup (100; *p* < 0.01) **(**Table 3**)**. No upregulation in AKR1C3 was found in either ADT-PCa subgroup.

**Table 3:** Expression of target proteins in the epithelial cells of the prostate tissue

Expressions of proteins	H-score cut-off^**a**^	Upregulated cancer tissues							
		Primary PCa (***n*** = 7)		ADT-PCa (***n*** = 10)					
				≤ 12 months (***n*** = 4)			> 12 months (***n*** = 6)		
		n	%	n	%	Compared to primary PCa^**b**^	n	%	Compared to primary PCa^**b**^
AR	57%	1	14	2	50	0.2	6	100	< 0.01*
AKR1C3	39%	2	29	**0**	**0**	0.23	1	17	0.61
SRD5A1	41%	5	71	2	50	0.47	4	67	0.85
SRD5A2	85%	3	43	2	50	0.81	3	50	0.79
SRD5A3	41%	7	100	1	25	< 0.01*	3	50	0.03*

^a^ H-Score cut-off was measured from mean H-score of BPH group (n = 6) as mentioned in the method, to determine upregulation of protein expressions in malignant tissues (Primary PCa and ADT ≤ 12 months and > 12 months)

^b^ Statistical significance was measured for p-value by comparing the H score of ADT ≤ 12 months and > 12 months with Primary PCa using Pearson Chi Square

^*^P-value < 0.05 is significant

### Associations between protein expression in the prostatic tissue of ADT-PCa patients and clinicopathological parameters

A further analysis of the ADT-PCa patients was performed by examining the association between increased protein expression and the clinical status of the patients (**data not shown**). This part of the study focused on the ADT-PCa subgroups. Specifically, we evaluated the ADT ≤12 months subgroup (Table 4) to determine whether the initial changes within the intraprostatic tissue were due to ADT. Half of the patients in the ADT ≤12 months group and all of the patients in the ADT > 12 months group had upregulated AR expression. Furthermore, one patient in the ADT ≤12 months subgroup had shown AR upregulation only 3 months after beginning ADT. However, there was no case of upregulation of AKR1C3 expression in the ADT ≤12 months subgroup. Additionally, there was no notable finding for SRD5A1, SRD5A2, and SRD5A3 protein expression. Moreover, there were no notable significant differences in the clinical or pathological parameters between the patients with upregulated and nonupregulated AR within the ADT ≤12 months subgroup.

**Table 4:** ADT-treated prostate cancer patients characteristics and clinical and pathological parameters

Characteristics	AR			SRD5A1			SRD5A2			SRD5A3		
	ADT < 12 Months		***P***-Value^b^	ADT < 12 Months		***P***-Value^b^	ADT < 12 Months		***P***-Value^b^	ADT < 12 Months		***P***-Value^b^
	High (***n*** = 2)	No (***n*** = 2)		High (***n*** = 2)	No (***n*** = 2)		High (***n*** = 2)	No (***n*** = 2)		High (***n*** = 1)	No (***n*** = 3)	
**Age**	59.5 ± 0.7	58 ± 5.6	0.75	56.5 ± 3.5	61 ± 1.4	1	56.5 ± 3.5	61 ± 1.4	1	54	60.3 ± 1.5	0.07
**T Stages**			0.05			0.32			0.32			0.25
1	–	–										
				–	–		–	–		–	–	
2	100%	–		50%	50%		50%	50%%		–	67%	
3	–	–		–	–		–	–		–	–	
4	–	100%		50%	50%		50%	50%		100%	33%	
**Gleason score**^**a**^			0.25			0.25			0.25			0.50
< 7	50%	–		–	50%		–	50%		–	33%	
08–10	50%	100%		100%	50%		100%	50%		100%	67%	
**PSA level**^**a**^	160.6 (17.9–303.3)	20.2 (10.1–30.4)	0.19	156.7 (10.1–303.3)	22.3 (17.9–30.4)	1	156.7 (10.1–303.3)	22.3 (17.9–30.4)	1	10.1	30.4 (17.9–303.3)	0.5
**ADT Type**			–			–			–			–
Orchyde	2	–		1	1		1	1		–	1	
LHRH agonist + antiandrogen												
	–	2		1	1		1	1		1	2	
**ADT Treatment Duration**	5 (3–7)	6 (3–9)	–	8 (7–9)	3	–	8 (7–9)	3	–	9	3 (3–7)	–

+ is upregulated protein expression group; − is downregulated protein expression group

^a^ Age is presented in mean ± SD; PSA level and prostate volume are presented in median (min-max)

^b^ Statistical significance was measured by comparing the clinical parameters of upregulated and non-upregulated ADT-PCa samples using Independent T test for Age; Pearson chi square test for Gleason score; Mann-Whitney test for T-stages and PSA level

^*^P-value < 0.05 is significant

Among the patients in the group given ADT for ≤12 months, one sample had increased protein expression of AR only, one sample had increased protein expression of SRDA1, 2, and 3 proteins, one sample increased protein expression of AR, SRDA1 and SRDA2, and one had no increase in protein expression. The specific H-scores for protein expression are available in Supplementary Table 2.

## Discussion

Although chemical castration by ADT has been the standard frontline therapy for advanced-stage PCa, disease progression is predicted to occur when cancer develops into CRPC. To better understand the changes involved in the transition to the progressive state, thorough study of the mechanisms of ADT resistance is important. The upregulation of components of the AR signaling pathway in intraprostatic tissue is one of the resistance mechanisms in CRPC. This study found upregulation of AR and steroidogenic enzymes in ADT-treated PCa patients. Furthermore, this study, in accordance with other studies, found that CRPC can develop in less than 12 months after the commencement of ADT [19] However, until now, no study has evaluated when the mechanism that triggers resistance to ADT becomes active in the prostate during ADT.

This study delves into the early response to ADT by evaluating intraprostatic AR and steroidogenic enzyme changes using prostate tissue from patients who still experienced urinary retention during ADT. It revealed a notably unique finding in the subgroup of patients who had ADT for only 12 months or less. Two patients had high intratumoral AR gene and protein expression after 3 months of ADT. It can be speculated that the resistance mechanism to ADT [10] through upregulation of AR might start as early as 3 months after the beginning of ADT. To the best of our knowledge, this is the first study to show early AR upregulation in human PCa tissue during ADT. This early resistance mechanism should be a warning to clinicians that this process should be monitored when starting ADT [20].

Another interesting result is that AR was the only gene that was upregulated at the early stage (3 months). PCa cells might start to overcome low serum androgen levels due to ADT by increasing AR expression first [10–13]. This suggests that the early mechanism to overcome low serum androgen levels is increased AR expression [10–13]. Many in vitro studies have shown upregulation of AR expression, demonstrating the adaptations of prostatic cells that increase sensitivity to low androgen levels after treatment with ADT [21–23]. However, these phenomena can be seen only in patients with orchiectomy who receive ADT. This might show that an abrupt decrease in serum testosterone levels induces the upregulation of AR. Furthermore, there are many known mechanisms of AR changes, including gene amplification and mutation, which have also been reported in patients with ADT > 12 months [8–10]. However, this study examined only protein expression and did not further evaluate the other AR changes, namely, AR amplification and AR mutation.

PCa cell growth is promoted by androgens, especially DHT [12]. This study found that 5α-reductase isoenzymes, which regulate the conversion of T to DHT [11, 12], were increased in ADT-treated PCa patients. Similar to other studies, SRD5A1 [14, 24] and SRD5A3 [14, 25] were upregulated in ADT-PCa patients compared with ADT-naïve PCa patients, and SRD5A2 was downregulated [14, 24, 26]. However, until now, there has been very limited information on whether the isozyme is involved in the process of androgen biosynthesis. Interestingly, this study found that SRD5A was the only steroidogenic enzyme that was upregulated in the ADT ≤12 months group. This SRD5A upregulation was also related to AR upregulation. This suggests that PCa cells upregulate the expression of SRD5A, which is the primary enzyme responsible for the conversion of T to DHT expression, after or at the same time as AR expression is upregulated [14]. Thus, developing a new strategy or compound that targets SRD5A can reduce the risk of early resistance.

Among the PCa-ADT patients with ADT durations of less than 12 months, three patients showed upregulation of genes with increased protein expression. Three of four patients showed upregulation of AR, with one patient showing upregulation AR and SRD5A1, 2, and 3; one showing upregulation of AR, SRD5A1 and SRD5A2; and one showing only AR upregulation. These findings are novel, as no one has ever investigated below the cut-off of 12 months. This study suggests the possible upregulation of AR and steroidogenesis enzymes (namely, SRD5A1, SRD5A2, and SRD5A3) as a compensatory mechanism for the low testosterone level due to ADT [14]. Our study showed that patients whose SRD5A1, 2, or 3 level becomes upregulated have increased expression of the proteins later (at 7–9 months of ADT) than those with upregulated AR (at 3–7 months of ADT). This suggests that the increase in AR is the first compensatory mechanism, followed by the increases SRD5A1, 2, and 3. However, further studies with more patients are needed to validate this compensatory response.

Many studies have shown that there is a shift to adrenal androgen usage for maintaining DHT levels via upregulation of AKR1C3 expression [10–14]. This study showed that AKR1C3 can only be found in patients treated with ADT for more than 12 months, which is in accordance with other studies [27]. The next question is why AKR1C3 is not upregulated in the early state. Based on the steroidogenic pathway, AKR1C3 is an upstream enzyme that converts adrenal androgen to downstream androgens, which are needed as a source of DHT [12–14]. To support the previous statement, we hypothesize that in PCa cells, AKR1C3 expression is increased after AR and SRD5A upregulation. However, it has been shown that there are many variations in AKR1C3, SRD5A and AR expression among patients in the ADT > 12 months group. This might be due to the dynamic process of steroidogenesis. The AR or steroidogenesis enzymes are regulated based on ‘real-time’ conditions as needed by PCa cells.

The main limitation of our study is the small sample size. However, this is the first study that tried to evaluate AR signaling pathway changes during ADT in human prostate tissues. This study also revealed an important finding in which PCa cells may adapt to low androgen levels caused by ADT before PSA levels rise. This finding is not the first significant one with interesting information that is limited by a low sample size. One study performed by Alsinnawi M et al. contributed significant prognostic information in which high expression of the SLCO gene may result in worse disease-free survival (DFS), with only 11 samples included in the study [28]. Although the sample size was small, the results of the mentioned study were in concordance with the results of Terakawa T et al.’s team, who examine similar outcomes but with more patients included (*n* = 494) [29]. Similar studies had similarly small sample sizes yet showed significance in practice. With only a few samples, Tiwari et al. showed that AR and its transcriptional corepressor REST modulate SPINK1 expression and that SPINK1 plays a plausible role in the progression of neuroendocrine prostate cancer [30]. Another study by Cheung et al., using only 11 samples per group, found that Actin alpha cardiac muscle 1 (ACTC1) gene expression plays a role in compensating ADT administration for PCa as a response to ADT-induced muscle loss [31].

In addition to the sample size, other limitations of our study include the limited number samples available due to some nonutilizable old specimens unsuitable for RNA extraction and protein expression evaluation. Another limitation of this study is its use of the median expression level of each gene in BPH tissues as the cut-off for defining upregulation in other samples. This was the only available method, as there is currently no official validated cut-off to define upregulation of AR or steroidogenic genes in immunohistochemistry staining.

In conclusion, AR and steroidogenic enzymes are upregulated in PCa patients who are treated with ADT. Early AR and SRD5A upregulation can be found at 3 months in ADT patients. This indicates that the early evaluation of AR and SRD5A expression in intraprostatic tissue should be done. Further strategic treatment should target AR and the SRD5A enzyme to overcome early resistance to ADT.

## Conclusion

AR and steroidogenic enzymes are upregulated in ADT-PCa patients as early as 3 months without PSA elevation. Steroidogenic enzymes, especially SRD5A3 expression, were also upregulated before PSA rose.

## Supplementary information

**Additional file 1: Supplementary Table 1.** Description of Antibodies used in the Study. **Supplementary Table 2.** H-score of the included samples.

## Data Availability

The datasets used and/or analysed during the current study are available in the supplementary tables while other related data may be accessible through the corresponding author upon reasonable request.

## Abbreviations

ACTC1: Actin alpha cardiac muscle 1
ADT: Androgen-Deprivation Therapy
AR: Androgen receptor
ARVs: Androgen receptor variants
BPH: Benign Prostatic Hyperplasia
CRPC: Castration resistant prostate cancer
DHT: Dihydrotestosterone
DFS: Disease-free survival
FFPE: Formalin Fixed Paraffin embedded
HIER: Heat induced Epitope retrieval
PCa: Prostate cancer
PSA: Prostate Specific Antigen
