# Supplementary material for: Early upregulation of AR and steroidogenesis enzyme expression after 3 months of androgen-deprivation therapy
Source: BMC Urol. 2020 Jun 19;20:71. doi: 10.1186/s12894-020-00627-0 (PMC7304221; doi:10.1186/s12894-020-00627-0)
Supplement: Supplementary file 1 — Additional file 1: Supplementary Table 1. Description of Antibodies used in the Study. Supplementary Table 2. H-score of the included samples. [file 12894_2020_627_MOESM1_ESM.docx]

**Supplementary table 1**. Description of Antibodies used in the Study

| **Protein target** | **Antibody product number** | **Biological source** | **Clone** | **Validation and reference** |
| --- | --- | --- | --- | --- |
| AR | WH0000367M1 | Mouse | Monoclonal | Peer reviewed^15,16^ |
| AKR1C3 | A6229 | Mouse | Monoclonal | Peer reviewed^15,17^ |
| SRD5A1 | HPA051402 | Rabbit | Polyclonal | Human Protein Atlas project (HPA) ^15^ |
| SRD5A2 | SAB2105567 | Rabbit | Polyclonal | - |
| SRD5A3 | HPA027006 | Rabbit | Polyclonal | Human Protein Atlas project (HPA) ^15^ |

**Supplementary table 2**. H-score of the included samples

| **Group** | **Sample number** | **Duration of ADT** | **AR** | **AKR3C1** | **SRD5A1** | **SRD5A2** | **SRD5A3** |
| --- | --- | --- | --- | --- | --- | --- | --- |
| Primary PCa | I | NA | 106.6 | 147.00 | 218.2 | 271.6 | 264.8 |
|  | II | NA | 80.4 | 99.00 | 188.4 | 243.4 | 229.8 |
|  | II | NA | 58.8 | 79.40 | 97.2 | 186.6 | 129.8 |
|  | IV | NA | 37.2 | 105.00 | 229 | 213.6 | 206.4 |
|  | V | NA | 48 | 68.80 | 185 | 253.8 | 191.6 |
|  | VI | NA | 75.8 | 92.80 | 83.6 | 188.6 | 162.8 |
|  | VII | NA | 214 | 210.60 | 222.2 | 286.2 | 283.2 |
| ADT under 12 | VIII | 3 | 133.4 | .80 | 84.8 | 98.6 | 13.2 |
|  | IX | 7 | 279 | 79.20 | 195.6 | 277.6 | 63.6 |
|  | X | 3 | 232.2 | 1.60 | 111.8 | 157.4 | 144 |
|  | XI | 9 | 98.4 | 10.40 | 161.4 | 273.8 | 151.2 |
| ADT above 12 | XII | 35 | 220.8 | 114.80 | 201.2 | 290.2 | 267.6 |
|  | XIII | 25 | 261.4 | 143.40 | 190 | 286.2 | 287.6 |
|  | XIV | 40 | 187.2 | 31.00 | 81 | 203.6 | 59.8 |
|  | XV | 15 | 174.2 | 14.00 | 70.8 | 206.8 | 38.6 |
|  | XVI | 70 | 238.6 | 7.40 | 170.8 | 188.6 | 140.8 |
|  | XVII | 24 | 247.8 | 19.60 | 166.4 | 259 | 110.8 |
| BPH | XVIII | NA | 173.6 | 136.60 | 165.4 | 271.4 | 199.2 |
|  | XIX | NA | 168 | 219.80 | 114.8 | 261.6 | 108.6 |
|  | XX | NA | 209 | 88.60 | 58.4 | 235 | 132 |
|  | XXI | NA | 150.8 | 86.00 | 141 | 249.8 | 118.2 |
|  | XXII | NA | 172.8 | 81.80 | 105.2 | 240.2 | 93.6 |
|  | XXIII | NA | 155 | 94.80 | 158.6 | 269.6 | 86.2 |
